# cFLIP downregulation is an early event required for endoplasmic reticulum stress-induced apoptosis in tumor cells

**DOI:** 10.1038/s41419-022-04574-6

**Published:** 2022-02-03

**Authors:** Rocío Mora-Molina, Daniela Stöhr, Markus Rehm, Abelardo López-Rivas

**Affiliations:** 1 Centro Andaluz de Biología Molecular y Medicina Regenerativa-CABIMER, CSIC-Universidad de Sevilla-Universidad Pablo de Olavide, Avda Américo Vespucio 24, 41092 Sevilla, Spain; 2 University of Stuttgart, Institute of Cell Biology and Immunology, Stuttgart, Germany; 3 Centro de Investigación Biomédica en Red-Oncología (CIBERONC), Carlos III Health Institute, Seville, Spain

**Keywords:** Cancer microenvironment, Apoptosis

## Abstract

Protein misfolding or unfolding and the resulting endoplasmic reticulum (ER) stress frequently occur in highly proliferative tumors. How tumor cells escape cell death by apoptosis after chronic ER stress remains poorly understood. We have investigated in both two-dimensional (2D) cultures and multicellular tumor spheroids (MCTSs) the role of caspase-8 inhibitor cFLIP as a regulator of the balance between apoptosis and survival in colon cancer cells undergoing ER stress. We report that downregulation of cFLIP proteins levels is an early event upon treatment of 2D cultures of colon cancer cells with ER stress inducers, preceding TNF-related apoptosis-inducing ligand receptor 2 (TRAIL-R2) upregulation, caspase-8 activation, and apoptosis. Maintaining high cFLIP levels during ER stress by ectopic expression of cFLIP markedly inhibits ER stress-induced caspase-8 activation and apoptosis. Conversely, cFLIP knockdown by RNA interference significantly accelerates caspase-8 activation and apoptosis upon ER stress. Despite activation of the proapoptotic PERK branch of the unfolded protein response (UPR) and upregulation of TRAIL-R2, MCTSs are markedly more resistant to ER stress than 2D cultures of tumor cells. Resistance of MCTSs to ER stress-induced apoptosis correlates with sustained cFLIP_L_ expression. Interestingly, resistance to ER stress-induced apoptosis is abolished in MCTSs generated from cFLIP_L_ knockdown tumor cells. Overall, our results suggest that controlling cFLIP levels in tumors is an adaptive strategy to prevent tumor cell’s demise in the unfavorable conditions of the tumor microenvironment.

## Introduction

Under environmental and physiological stress conditions that increase a load of unfolded or misfolded proteins in the endoplasmic reticulum (ER), protein sensors located in the luminal face of the ER membrane activate the unfolded protein response (UPR) to allow adaptive mechanisms that re-establish proteostasis [1]. However, unresolved ER stress results in the activation of cell death by apoptosis [2]. Upregulation of pro-apoptotic proteins and downregulation of antiapoptotic proteins of the Bcl-2 family have been observed in cells undergoing apoptosis upon ER stress [2–4]. Moreover, upregulation of TNF-related apoptosis-inducing ligand receptor 2 (TRAIL-R2) expression and its intracellular activation in a TRAIL-independent manner lead to TRAIL-R2-mediated extrinsic apoptotic pathway following ER stress [5–7]. More recently, it was demonstrated that misfolded proteins directly bind to and activate TRAIL-R2 in the ER–Golgi intermediate compartment to induce caspase-8 activation and apoptosis [8]. In addition, ER stress-promoted upregulation of TRAIL-R2 sensitizes tumor cells to TRAIL-induced apoptosis in two-dimensional cultures as well as in multicellular tumor spheroids (MCTSs) [9, 10].

Activation of TRAIL receptors by extracellular TRAIL leads to the formation of a death-inducing signaling complex (DISC), which includes mainly the receptor itself, the adapter molecule FADD and procaspase-8 [11]. Processing and activation of caspase-8 at the DISC lead to a cascade of apoptotic events which results in the death of the cell. At the DISC level, apoptosis signaling is regulated by cellular FLICE-Like Inhibitory Protein (cFLIP), the homolog of vFLIP in vertebrate cells [12]. Suppression of cFLIP expression in tumor cells induces caspase-8-dependent apoptosis both in vitro and in vivo and sensitizes these cells to TRAIL [13–15]. However, the role of cFLIP proteins in the control of the apoptotic response of tumor cells undergoing chronic ER stress has not been investigated.

Given the importance of cFLIP proteins in the control of DISC-mediated caspase-8 activation upon extracellular TRAIL binding to TRAIL receptors, we have examined the role of cFLIP as a modulator of the protein-folding checkpoint in tumor cells under irreversible ER stress conditions. Our findings show that cFLIP proteins downregulation is an early event involved in apoptosis induced upon ER stress in colon tumor cells growing in conventional 2D cultures. Remarkably, cFLIP_L_ downregulation induced by ER stress is strongly inhibited in tumor cells growing as 3D multicellular spheroids. Moreover, when compared with 2D cultures, spheroids show marked resistance to ER stress-induced apoptosis despite upregulation of TRAIL-R2/DR5. Importantly, cFLIP_L_ knockdown restored sensitivity to ER stress in tumor spheroids. Collectively, these results reveal that cFLIP_L_ levels play a key role to control TRAIL-R2-mediated caspase-8 activation and apoptosis in colon cancer cells undergoing ER stress.

## Results

### cFLIP protein levels and caspase-8 activation upon ER stress in tumor cells

In different two-dimensional (2D) tumor cell systems, sustained ER stress by misfolded or unfolded protein accumulation in the ER induces the PERK/ATF4/CHOP/TRAIL-R2/DR5-dependent and TRAIL-independent intracellular DISC assembly that activates a caspase-8-mediated apoptotic pathway [5–7]. While cFLIP_S_ impairs caspase-8 activation at the TRAIL DISC in the plasma membrane [16] it was recently reported that the function of cFLIP_L_ in TRAIL-induced apoptosis depends on its proportion in relation to caspase-8 at the DISC [17], as was also described in the CD95 system [18]. High levels of cFLIP_L_ block caspase-8 activation, but low levels of cFLIP_L_ promote its activation at the DISC. However, it remains unknown if cFLIP could also play a role in the regulation of TRAIL-R2/DR5-dependent caspase-8 activation and apoptosis in tumor cells undergoing ER stress.

**Fig. 1:**
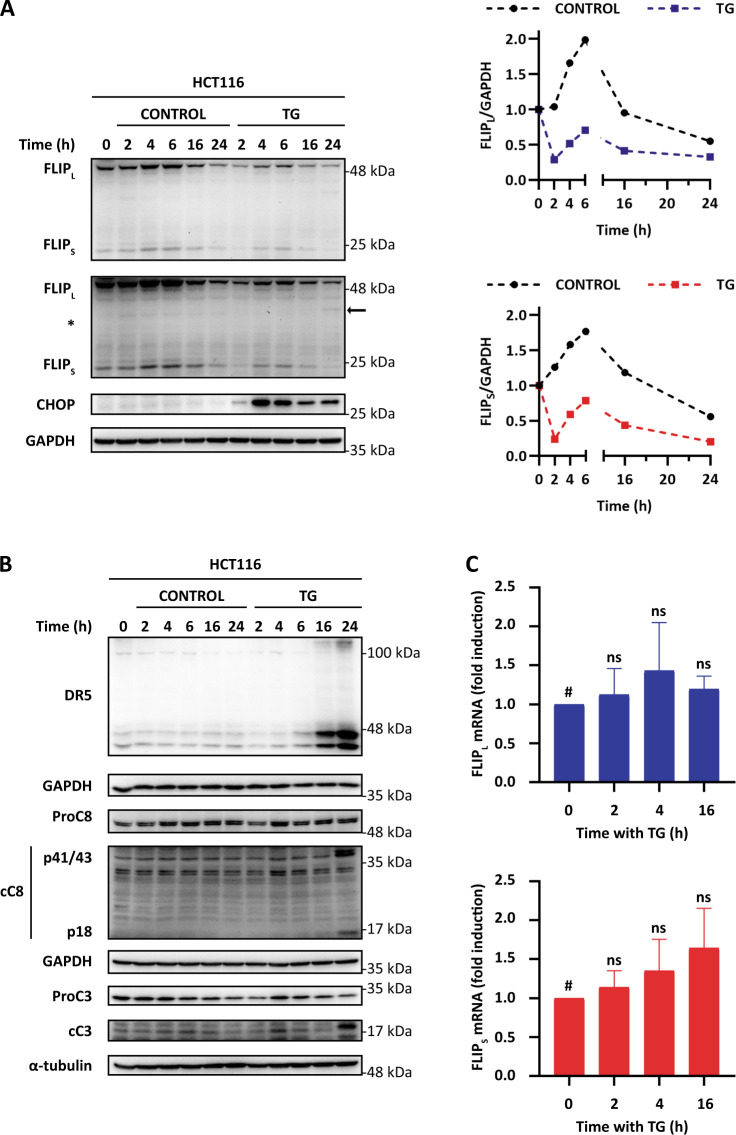
cFLIP levels and caspase-8 activation upon ER stress in tumor cells. HCT116 cells were treated with TG (100 nM) for the indicated times. cFLIP and CHOP levels (**A**) as well as TRAIL-R2/DR5 upregulation, caspase-8 and caspase-3 processing (**B**) were determined in whole-cell extracts by western blotting. Levels of both cFLIP isoforms were quantified with Image Lab^TM^ 6.0 software using GAPDH as protein-loading control and graphed relative to time 0 levels. Blots are representative of three independent experiments. In **A**, two different exposures of the western blot are shown to follow the levels of the short isoform of cFLIP. **C** HCT116 cells were treated with TG (100 nM) for the indicated times and mRNA relative levels of cFLIP_L_ (upper panel) and cFLIP_S_ (lower panel) were examined by RT-qPCR as described in materials and methods and referred to time 0 h levels (ns = not statistically significant. Unpaired *t* test with Welch’s correction).

To investigate the function of cFLIP proteins in ER stress-induced apoptosis we first examined cFLIP levels upon ER stress in 2D cultures of HCT116 colorectal cancer cells. HCT116 cells were treated with the ER stress inducer thapsigargin (TG) for the indicated times and cFLIP protein levels and caspase-3 processing were determined. TG treatment induced an early decline in cFLIP levels (Fig. 1A), long before caspase-3 activation could be observed (Fig. 1B). Downregulation of both cFLIP isoforms in HCT116 cells took place as early as 1 h after TG addition to cultures of HCT116 cells (Fig. S1). Likewise, treatment of HCT116 cells with tunicamycin, another ER stress-inducing agent, also resulted in a decrease in cFLIP proteins levels, although with slightly different kinetics (Fig. S1). Importantly, cFLIP down-regulation upon ER stress preceded the upregulation of TRAIL-R2/DR5 expression and the onset of caspase-8 activation as indicated by the processing of both caspase-8 and the long isoform of cFLIP (Fig. 1A, B). Early cFLIP downregulation in the extrinsic apoptotic pathway activated upon TG treatment was also observed in p53-null HCT116 and HT-29 (mutant p53) cell lines (Figs. S2 and S3) indicating that ER stress-induced cFLIP loss upon ER stress is not limited to HCT116 cell line and is independent of p53 status.

Activation of the PERK branch of the UPR upon ER stress leads to the expression of the transcription factor DDIT3/CHOP (Fig. 1A) which is responsible for the upregulation of TRAIL-R2/DR5 in different cell types [5–7]. Interestingly, expression of the DDIT3/CHOP gene in HCT116 cells was first detected at 2–4 h of treatment with the ER stress inducer (Fig. 1A and S1). Together, these results indicate that cFLIP downregulation is one of the earliest events observed in the signaling pathway leading to caspase-8 activation in tumor cells undergoing ER stress.

**Fig. 2:**
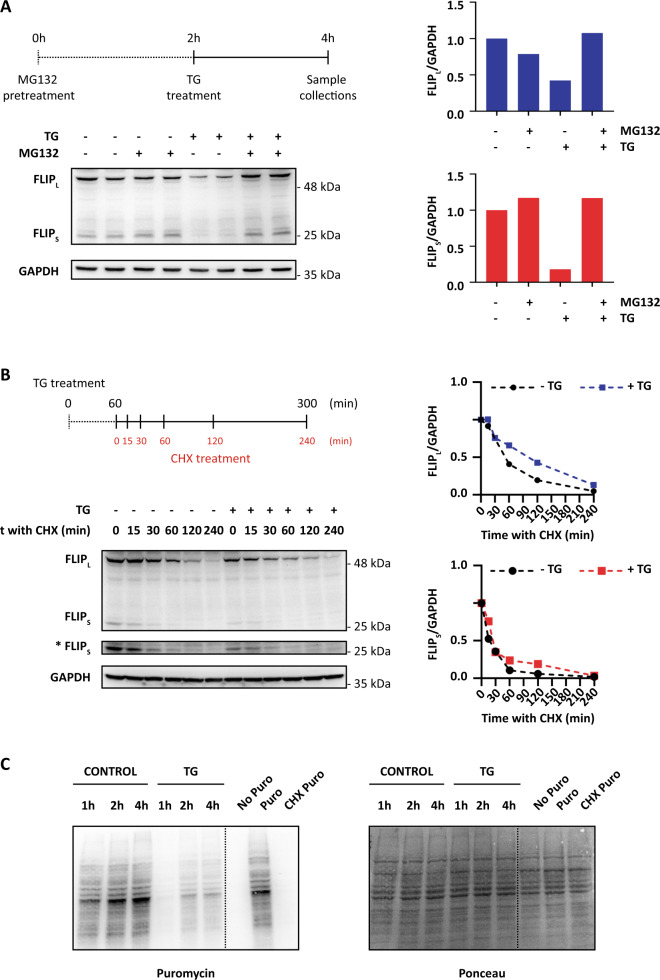
Proteosomal degradation of FLIP proteins and inhibition of global protein synthesis upon ER stress in tumor cells. **A** HCT116 cells were treated with or without MG-132 (20 μM) for 2 h and then TG (100 nM) was added or not for a further 2-h period. cFLIP levels were determined in whole-cell extracts by western blotting. Levels of both cFLIP isoforms were quantified using GAPDH as protein-loading control through Image Lab^TM^ 6.0 software. Blots are representative of three independent experiments. **B** To analyze cFLIP stability under ER stress, HCT116 cells were treated or not with TG (100 nM) for 2 h prior to treatment with cycloheximide (CHX) (5 μg/mL) for the indicated times. cFLIP levels were determined in whole-cell extracts by western blotting. Levels of cFLIP_L_ were quantified using GAPDH as protein-loading control through Image Lab^TM^ 6.0 software and referred to time 0 h levels. Blots are representative of two independent experiments. A longer exposure time is also included (*) to determine cFLIPs stability. **C** HCT116 cells were treated with or without TG (100 nM) for the indicated times and puromycin (1 μg/mL) was added for the last 10 min of treatment. Puromycin incorporation to the nascent protein chain was assessed by western blotting using an anti-puromycin antibody as described under Materials and Methods. As controls of efficiency of puromycin detection in testing protein synthesis, puromycin was or not added or added in the last 10 min of a 1h-CHX treatment. Ponceau staining was used to test the same amount of protein was present in each lane. Blots are representative of three independent experiments.

To get further insight into the mechanism underlying the loss of cFLIP proteins expression in 2D cultures of HCT116 cells treated with TG, we first determined by RT-qPCR the mRNA levels of both cFLIP_L_ and cFLIP_S_. Results shown in Fig. 1C indicate that in contrast to the observed down-regulation of cFLIP protein levels following TG treatment, mRNA levels did not decrease at any of the time points examined. We next investigated whether protein degradation through the ubiquitin-proteasome pathway could be responsible for the decline in cFLIP protein levels upon ER stress. As shown in Fig. 2A, treatment of HCT116 cells with the proteasome inhibitor MG-132 blocked TG-induced down-regulation of both cFLIP isoforms, which indicates that proteasomal degradation of cFLIP is involved in the down-regulation of cFLIP levels upon ER stress. As both cFLIP isoforms are short-lived proteins subject to ubiquitination and degradation by the proteasome in tumor cells treated with different anti-tumor drugs, we then examined whether TG treatment might decrease their half-lives in HCT116 cells. However, results shown in Fig. 2B demonstrate that under conditions of translation inhibition differential half-times/stabilities of cFLIP species could no longer be observed between control and TG-treated cell populations.

During ER stress, phosphorylation of eukaryotic translation initiation factor eIF2α by PERK results in a reduction of general protein synthesis and thus a decrease in the load of proteins entering the ER. Since neither cFLIP mRNAs levels nor proteins half-lives were reduced in cells undergoing ER stress we determined the kinetics of eIF2α phosphorylation and protein synthesis inhibition upon TG treatment. As shown in Fig. 2C, protein synthesis was markedly inhibited in HCT116 cells as early as 1 h after the addition of the ER stress inducer to the cultures, closely resembling the kinetics of eIF2α phosphorylation and cFLIP down-regulation (Fig. S1). Similar results were obtained in experiments where tunicamycin was used as ER stress-inducing agent, although with slightly different kinetics (Fig. S1). The expression of Mcl-1, another short-lived protein, was also rapidly downregulated in HCT116 cells upon TG treatment further supporting the hypothesis that inhibition of translation upon ER stress was responsible for the downregulation of these short-lived proteins (Fig. S1). To further demonstrate the importance of protein synthesis inhibition in the downregulation of cFLIP expression upon ER stress the effect of the integrated stress response inhibitor (ISRIB) on general protein synthesis and cFLIP levels was determined. As reported in other cell types [19], pre-treatment of HCT116 cells with ISRIB reversed the effect of TG on translation (Fig. S4A). Interestingly, there was a marked inhibition of ER stress-induced cFLIP loss in those cultures treated with ISRIB (Fig. S4B). Together, all these results suggest that cFLIP loss in 2D cultures of HCT116 cells upon ER stress is most likely a consequence of the reduced activity of the protein synthesis machinery and the proteasomal degradation of the remaining protein.

### Effect of ectopic expression or knockdown of cFLIP on ER stress-induced caspase-8 activation and apoptosis

**Fig. 3:**
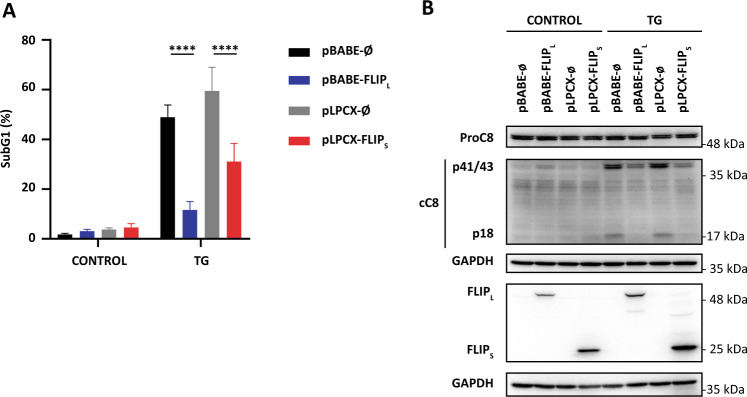
Effect of ectopic expression of cFLIP on ER stress-induced caspase-8 activation and apoptosis. HCT116 pBABE-ø, pBABE-cFLIP_L_, pLPCX-ø, and pLPCX-cFLIP_S_ cells were treated or not with TG (100 nM). **A** Apoptosis was analyzed after 30 h treatment by subG1 analysis as described under Materials and Methods (****p ≤ 0.0001; two-way ANOVA. Tukey’s multiple comparisons test). **B** cFLIP levels and caspase-8 processing were determined in whole-cell extracts by western blotting after 24-h treatment. GAPDH was used as a protein loading control. Blots are representative of three independent experiments.

To further explore the role of cFLIP in the induction of apoptosis upon ER stress we first enforced expression of ectopic cFLIP_L_ or cFLIP_S_ in HCT116 cells and then analyzed their response to TG. To this end, HCT116 cell lines stably expressing ectopic cFLIP_L_ or cFLIP_S_ were generated by infecting HCT116 cells with retroviruses carrying vectors for cFLIP_L_ or cFLIP_S_. As shown in Fig. S5A, both cell lines were highly resistant to activation of the extrinsic apoptotic pathway by TRAIL. We then examined the activation of apoptosis upon ER stress in cells ectopically expressing cFLIP. Marked inhibition of TG-induced apoptosis (Fig. 3A) was observed in HCT116-cFLIP_L_ cells as compared to cells infected with the control vector. Likewise, as shown in Fig. 3A, HCT116-cFLIP_S_ cells were also resistant to TG. Furthermore, in agreement with the antiapoptotic function of cFLIP proteins, caspase-8 processing upon TG treatment was clearly inhibited in both HCT116-cFLIP_L_ and HCT116-cFLIPs cell lines (Fig. 3B).

**Fig. 4:**
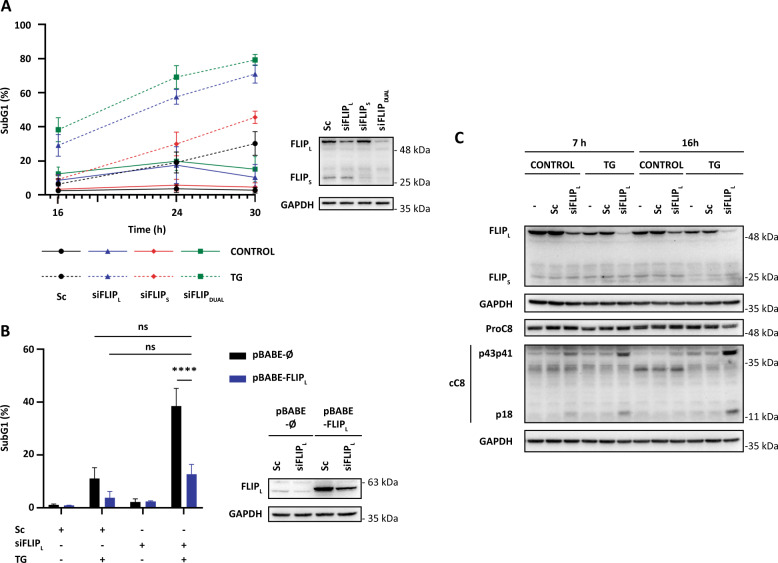
Effect of cFLIP knockdown on ER stress-induced caspase-8 activation and apoptosis. **A** HCT116 cells were transfected with scrambled oligonucleotide (Sc) or FLIP_L_, FLIP_S_, or FLIP_DUAL_ siRNAs for 48 h prior to TG (100 nM) treatment for the indicated times and apoptosis was determined by subG1 analysis. cFLIP knockdown was assessed in whole-cell extracts by western blotting. GAPDH was used as protein loading control. **B** HCT116 pBABE-ø or pBABE-cFLIP_L_ cells were transfected for 48 h with scrambled oligonucleotide (Sc) or FLIP_L_ siRNAs and apoptosis was determined after a further 24 h of thapsigargin treatment (ns = not statistically significant; ****p ≤ 0.0001; two-way ANOVA. Tukey’s multiple comparisons test). cFLIP_L_ levels were assessed by western blotting. **C** HCT116 cells were either non-transfected (−) or transfected with scrambled oligonucleotide (Sc) or cFLIP_L_ siRNA for 48 h prior to TG treatment for the indicated times. cFLIP protein levels and caspase-8 processing were determined in whole-cell extracts by western blotting. GAPDH levels were used as protein loading control. Blots are representative of three independent experiments.

The role of cFLIP levels in the onset of ER stress-induced cell death was also assessed by determining the effect of the specific down-regulation of cFLIP proteins by RNA interference in TG-induced apoptosis. Silencing cFLIP_L_ expression by siRNA prior to TG treatment strongly sensitized HCT116 cells to TG-induced apoptosis (Fig. 4A). Sensitization to ER stress-induced apoptosis was also observed upon silencing cFLIP_S_ expression although in this case, the percentage of apoptotic cells was markedly lower than the one observed after cFLIP_L_ knockdown (Fig. 4A). Moreover, simultaneous knockdown of both cFLIP_L_ and cFLIP_S_ (siFLIP_DUAL_) caused a stronger sensitization of HCT116 cells to TG-induced apoptosis as compared to the results obtained with each siRNA separately (Fig. 4A). Importantly, sensitization by cFLIP_L_ siRNA was specific to cFLIP_L_ knockdown as it was prevented in cells ectopically over-expressing cFLIP_L_ (Fig. 4B). Furthermore, cFLIP_L_ down-regulation by siRNA markedly accelerated caspase-8 processing upon TG treatment (Fig. 4C).

Neither activation of the PERK branch of the UPR, assessed by ATF4 and CHOP protein induction, nor up-regulation of TRAIL-R2/DR5 by TG were affected by ectopic cFLIP_L_ expression (Fig. S5B). These results suggest that maintaining high cFLIP levels in HCT116 cells undergoing ER stress is sufficient to prevent the activation of a caspase-8-dependent apoptotic program downstream of the upregulation of TRAIL-R2/DR5. Furthermore, we also examined the effect of cFLIP_L_ knockdown on the activity of the PERK branch of the UPR upon ER stress. As shown in Supplementary Fig. S6A silencing cFLIP_L_ expression did not result in an alteration of the levels of the PERK pathway markers when compared to cells transfected with a control oligonucleotide. Furthermore, TRAIL-R2/DR5 upregulation induced by TG was not affected by silencing cFLIP_L_ expression (Fig. S6A).

Collectively, these results demonstrate that the activation of caspase-8 and apoptosis upon ER stress clearly depend on the levels of cFLIP_L_, with a minor role of cFLIP_S_ in this scenario. Moreover, they also reveal that the effects derived from sustained cFLIP expression or knockdown reside downstream the activation of the PERK branch of the UPR and the up-regulation of TRAIL-R2, most likely by controlling the activation of caspase-8 at the intracellular DISC formed upon ER stress [5, 8].

### Role of cFLIP in the delayed activation of apoptosis in MCTSs upon ER stress

**Fig. 5:**
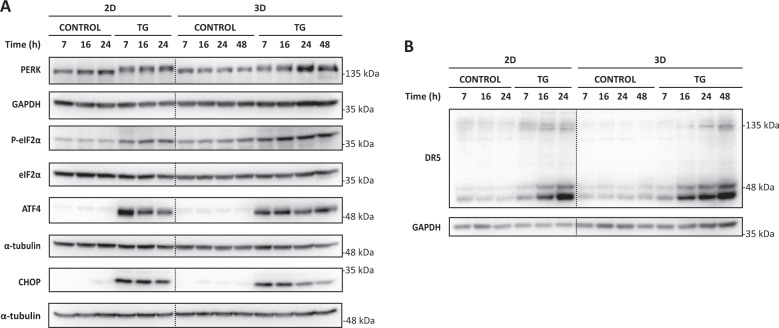
PERK pathway activation upon ER stress in bidimensional cultures and multicellular tumor spheroids. HCT116 cells, in conventional 2D cultures or spheroids (3D) (10-days), were treated with TG (100 nM) for the indicated times. Activation of the PERK signaling pathway (**A**) and TRAIL-R2/DR5 protein levels (**B**) were assessed in whole-cell extracts by western blotting. α-tubulin or GAPDH were used as protein-loading controls.

Solid tumors grow in a three-dimensional (3D) spatial conformation that enhances intercellular communication and allows the activation of adaptive responses to overcome the various types of inherent stresses in the 3D architecture of tumors [20, 21]. In this regard, it has been shown that compared to 2D cultures, in vitro cultures of MCTSs better resemble the 3D architecture of growing tumors [22]. To investigate further the function of cFLIP in the apoptotic response of tumor cells to ER stress, we first analyzed the activation of the pro-apoptotic PERK branch of the UPR in both 2D and MCTS cultures of HCT116 human colon cancer cells upon treatment with TG. As shown in Fig. 5A, phosphorylation of the PERK substrate eIF2α was stimulated by TG treatment in both 2D cultures and MCTSs. Likewise, no marked differences were observed between 2D and 3D cultures in the induction of ATF4 and CHOP transcription factors following treatment (Fig. 5A). Importantly, TRAIL-R2/DR5 upregulation, a limiting event in ER stress-induced apoptosis in 2D cultures [5–7], was not significantly impeded in MCTSs treated with TG (Fig. 5B).

**Fig. 6:**
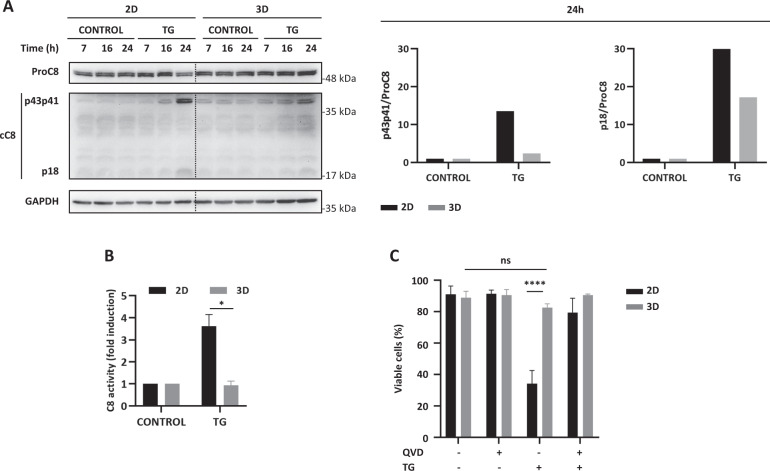
Delayed activation of apoptosis in multicellular tumor spheroids upon ER stress. **A** Cultures of HCT116 cells growing in 2D or as spheroids (10-days) were treated with TG (100 nM) for the indicated times. Caspase-8 processing was determined in whole-cell extracts by western blotting. Caspase-8 processing at 24 h of TG treatment was quantified using GAPDH as protein-loading control with Image Lab^TM^ 6.0 software and graphed relative to 2D- or 3D-untreated controls. Blots are representative of three independent experiments. **B** 2D or 3D cultures of HCT116 cells were treated with or without TG (100 nM) for 24 h and caspase-8 activity determined by an enzymatic specific assay as described in “Material and methods” and graphed relative to 2D- or 3D-untreated conditions (*p ≤ 0.05. Multiple *t*-test. Holm–Sidak method). **C** 2D or 3D cultures of HCT116 cells were treated with or without TG (100 nM) in the presence or absence of pan-caspase inhibitor Q-VD-OPh (20 μM) for 3 days. Cell viability was analyzed after staining with Annexin V-FITC and PI by flow cytometry (ns = not statistically significant; ****p ≤ 0.0001; two-way ANOVA. Tukey’s multiple comparisons test).

**Fig. 7:**
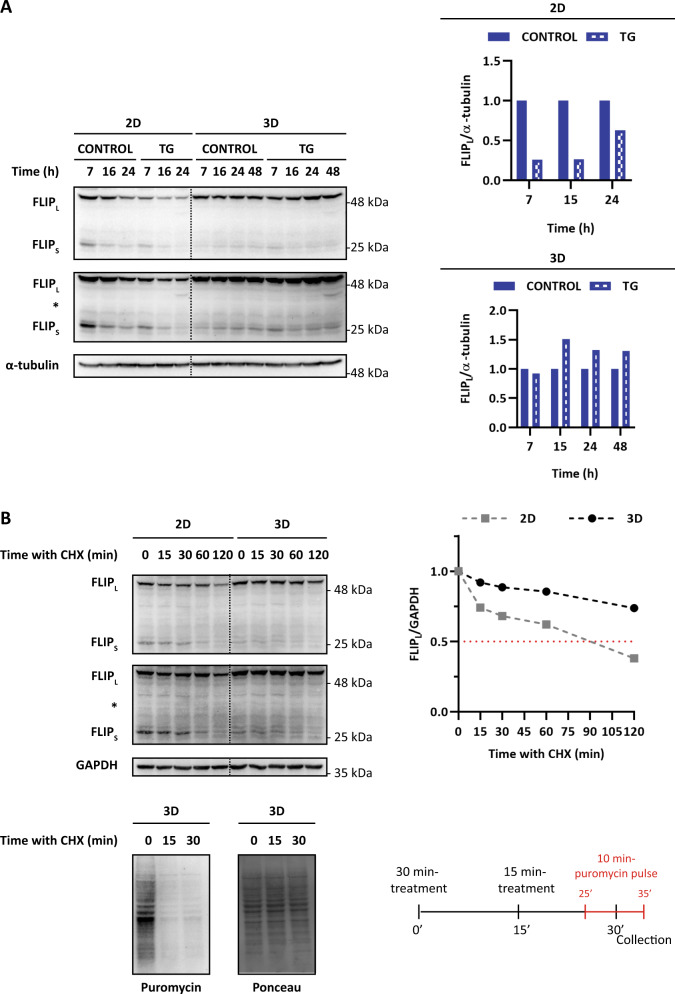
Role of cFLIP in the delayed activation of apoptosis in multicellular tumor spheroids upon ER stress. **A** Cultures of HCT116 cells growing in 2D or as spheroids (10-days) were treated with TG (100 nM) for the indicated times. cFLIP_L_ levels were determined in whole-cell extracts by western blotting. Quantification of FLIP loss after ER stress was performed using Image Lab^TM^ 6.0 software by taking its respective untreated cultures as control at each time point. **B** To compare cFLIP_L_ stability, 2D and 3D cultures were treated with CHX (5 μg/mL) for the indicated times (upper panel). cFLIP levels were determined in whole-cell extracts by western blotting. Levels of cFLIP_L_ were quantified using GAPDH as protein-loading control through Image Lab^TM^ 6.0 software and referred to time 0 h levels. As control of efficiency of CHX treatment in inhibiting protein synthesis in 10-day-old HCT116 spheroids (3D), puromycin was added for 10 min and its incorporation was determined in whole-cell extracts by western blotting as indicated in the scheme (lower panel). Ponceau staining confirms that the same amount of protein was present in each lane.

Even though there were no significant differences in TRAIL-R2/DR5 upregulation between 2D and MCTSs upon treatment with TG (Fig. 5B), caspase-8 processing (Fig. 6A) and activity (Fig. 6B) as well as induction of apoptosis (Fig. 6C) were all substantially inhibited in spheroids from HCT116 cells as compared to 2D cultures. Indeed, a slight activation of apoptosis was only observed after 4-days treatment of MCTSs with TG (Fig. S6B). We then analyzed the cellular levels of cFLIP proteins after treatment with TG at different times. Results depicted in Fig. 7A clearly demonstrate that, in contrast to the marked down-regulation of both cFLIP isoforms observed in 2D cultures of HCT116 cells treated with TG, cFLIP_L_ levels remained elevated in MCTSs up to 48 h after addition of the ER stress inducer.

Importantly, although there were no significant differences in the activation of the PERK branch of the UPR and TRAIL-R2/DR5 up-regulation (Figs. S7A, B and S8A, B), downregulation of cFLIP_L_ expression and caspase-8 activity upon ER stress were also markedly inhibited in spheroids of p53-null HCT116 and HT-29 cells (Figs. S7C, D and S8C, D).

Analysis of protein half-life in the presence of cycloheximide indicated increased stability of cFLIP_L_ protein in MCTSs as compared to 2D cultures of HCT116 cells (Fig. 7B). Unlike what is observed with cFLIP_L_, the basal levels of cFLIPs in HCT116 cells growing in spheroids were considerably lower than the levels observed in monolayer cultures of these cells (Fig. 7A, B). This fact precluded a reliable analysis of the effect of ER stress on cFLIPs expression in 3D cultures. When compared to 2D cultures of colon cancer cells, spheroids exhibit a marked inhibition in various signaling pathways and reduced cell cycle progression [23]. In particular, mTORC1 activity is substantially inhibited in tumor spheroids [23]. Results in figure S9A show that spheroids of HCT116 cells displayed markedly inhibited phosphorylation of the mTORC1 substrate 4EBP1 compared to 2D cultures. To assess the impact that inhibition of mTORC1 activity may have on ER stress-induced changes in cFLIP levels and apoptosis in HCT116 cells, 2D cultures were treated with either rapamycin or Torin-1 prior to incubation in the presence of TG. Inhibiting mTORC1 significantly increased cFLIP_L_ levels in HCT116 cells (Fig. S9B). Furthermore, the addition of mTORC1 inhibitors to cultures of HCT116 cells contributed to maintaining higher cFLIP_L_ levels in TG-treated cells and reduced TG-induced caspase-8 processing and apoptosis in 2D cultures of HCT116 cells (Fig. S9B, C). Collectively, these results suggest that by keeping a reduced mTORC1 activity, tumor cells in spheroids might be protected from ER stress-induced cFLIP_L_ loss and apoptosis, although the precise mechanism underlying the increased stability of cFLIP_L_ protein and the inhibition of cFLIP_L_ down-regulation upon TG treatment in MCTSs remains to be characterized.

**Fig. 8:**
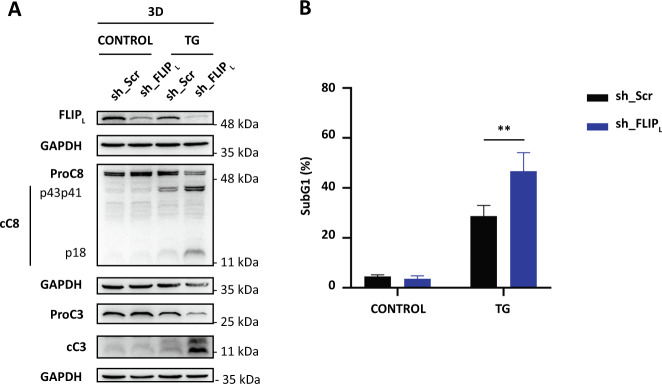
Effect of cFLIP_L_ knockdown on ER stress-induced apoptosis in multicellular tumor spheroids. Spheroids (10 days) of HCT116 shScr or shFLIP_L_ cells were treated with TG (100 nM) for 3 days. **A** cFLIP_L_ levels and processing of caspase-8 and -3 were assessed in whole-cell extracts by western blotting using GAPDH as protein-loading control. Blots are representative of three independent experiments. **B** Apoptosis was determined following TG treatment by subG1 analysis as described under “Materials and methods” (**p ≤ 0.005; two-way ANOVA. Tukey’s multiple comparisons test).

Together, all these results support the proposition that cFLIP_L_ loss is an early event required for the activation of extrinsic apoptosis in colon tumor cells undergoing ER stress that is inhibited in spheroids. To address this, we stably silenced cFLIP_L_ expression in HCT116 cells to examine the sensitivity of MCTSs to ER stress. Stable cFLIP_L_ knockdown sensitized HCT116 cells growing in 2D cultures to both TRAIL- and TG-induced apoptosis (Fig. S10A) and caspase-8 activation (Fig. S10B), which confirmed data obtained with siRNAs (Fig. 4). Next, we determined the apoptotic response to ER stress in MCTSs generated from cFLIP_L_ knockdown HCT116 cells. Results shown in Fig. 8 demonstrate that reducing cFLIP_L_ expression significantly sensitizes MCTSs to ER stress-induced caspases activation (Fig. 8A) and apoptosis (Fig. 8B). As tumor spheroids more closely reproduce different features of solid tumors, our data also suggest that intratumor rewiring of adaptive responses to microenvironmental stress may help to prevent the activation of an apoptotic process by maintaining cFLIP levels. Therefore targeting cFLIP may represent a valid anti-tumor strategy as recently proposed [24, 25].

## Discussion

Uncontrolled proliferation of malignant cells in growing tumors results in the generation of different stressors in the tumor microenvironment such as nutrient shortage, hypoxia and acidosis, among others, which disrupt ER homeostasis and cause persistent ER stress [21]. In conventional 2D cultures of tumor cells, sustained ER stress has been shown to activate the extrinsic apoptotic pathway through PERK pathway-mediated upregulation of TRAIL-R2/DR5 expression that induces the activation of caspase-8 at an intracellular DISC [5–8]. However, despite the importance of cFLIP levels in controlling the extrinsic pathway of apoptosis triggered upon TRAIL-R2/DR5 activation by its ligand [13, 14], the role of cFLIP in cell fate decisions upon ER stress remained unknown.

Both in vitro and in vivo studies have indicated a survival role of cFLIP in the viability of colon cancer cells inhibiting chemotherapy-induced apoptosis [26]. Our results demonstrate that TRAIL-R2/DR5 up-regulation and apoptosis in 2D cultures of colon tumor cells undergoing ER stress is preceded by an early decrease in the expression levels of cFLIP proteins, which alters the caspase-8/FLIP ratio facilitating caspase-8 activation and apoptosis, as recently demonstrated in TRAIL-induced apoptosis [16, 17]. Interestingly, in addition to the canonical role of cFLIP spliced isoforms as regulators of DISC-dependent caspase-8 activation at the plasma membrane, it was recently reported that cFLIP_L_ localizes to the ER in MEFs where it was shown to inhibit caspase-8-mediated cleavage of an ER-localized protein substrate [27].

Collectively, our results reveal a key role of cFLIP_L_ in maintaining cell viability under ER stress in colon cancer cells. This is in contrast to a recent study where cFLIP deletion in mouse embryonic fibroblasts (MEFs) was associated to reduce the activity of the PERK and Ire-1α UPR pathways and increased resistance to apoptosis upon ER stress [28]. At present, we do not know if the different role of FLIP in the response to ER stress is due to differences in UPR signaling between tumor and non-tumor cells. Alternatively, long-term cFLIP ablation in MEFs may have caused these cells to adopt a rewired ER stress-responsive survival pathway.

Our data point to the deregulation of the mechanisms controlling cFLIP_L_ levels [29] in 3D cultures of tumor cells, as an essential event in the process leading to apoptosis inhibition under chronic ER stress. Previous studies have indicated that cell morphology and intracellular signaling pathways are markedly altered in 3D cultures as opposed to conventional monolayer cultures of tumor cells [23, 30]. Our results demonstrate that the PI3K-AKT-mTORC1 pathway is significantly inhibited in spheroids as previously reported [23] and could play a role in the control of cFLIP_L_ levels and apoptosis upon ER stress. In addition, the decline in cell cycle progression resulting from inhibition of signaling pathways in spheroids [23] may also contribute to maintaining cFLIP levels [31] thus conferring resistance to ER stress-induced activation of the TRAIL-R2/DR5/caspase-8 pathway of apoptosis.

Our findings also reveal that in tumor spheroids, which more closely mimic the properties of solid tumors [32], cFLIP_L_ levels remained high during ER stress despite activation of the PERK/ATF4/CHOP branch of the UPR and up-regulation of TRAIL-R2/DR5 expression. Interestingly, maintenance of cFLIP_L_ levels in tumor spheroids was associated with increased cFLIP_L_ protein stability and resistance to ER stress-induced apoptosis. cFLIP proteins are short-lived inhibitory proteins subjected to rapid turnover regulated by the ubiquitin–proteasome system [33]. Different ubiquitin E3 ligases have been identified as being responsible for the degradation of cFLIP proteins by the proteasome [34–36]. Furthermore, the expression of ubiquitin E3 ligases targeting cFLIP for degradation is down-regulated in gastric and colorectal cancer [37, 38]. Importantly, elevated levels of cFLIP isoforms have been observed in tumor samples from different cancers, including colorectal tumors, which suggests a protumoral role of this inhibitor of the extrinsic apoptotic pathway [39–41]. In particular, high cFLIP_L_ levels have been found to correlate with poor prognosis in colorectal cancer patients [42]. In this context, our results suggest that cFLIP_L_ levels may play a key role in cell fate decisions under stress as maintenance of FLIP_L_ levels will prevent early activation of the death receptor-activated apoptotic pathway. This will provide time for the tumor cells to mount an adaptive response to the stressful conditions of the tumor microenvironment thus allowing tumor growth and progression. On the other hand, this dependence of colon tumor cells on the maintenance of FLIP levels in stressful situations to maintain cell viability reveals a vulnerability of these cells that could be used as a target for therapeutic intervention in colon cancer.

## Materials and methods

### Cell culture

Human colorectal carcinoma cell line HCT116 (a donation of Dr. J.A. Pintor-Toro, CABIMER, Seville, Spain) was originally obtained from the American Type Culture Collection. HCT116 p53^−/−^ cell line was a kind gift from Dr. Bert Vogelstein (Johns Hopkins University, Baltimore, MD). HT-29 colon cancer cell line was obtained from Cell Lines Service GmbH (CLS, Germany). Cell lines were regularly tested for mycoplasma contamination. All cell lines were cultured in McCoy’s 5A modified medium, supplemented with 2 mM l-glutamine, penicillin (50 U/ml), streptomycin (50 μg/ml), and 10% fetal bovine serum. Cells were grown at 37 °C, in a 5% CO_2_-humidified, 95% air incubator.

### Reagents and antibodies

Soluble human His-tagged recombinant TRAIL was generated in our laboratory as described [43]. Tunicamycin, thapsigargin, cycloheximide, puromycin, and proteasome inhibitor MG132 were from Sigma-Aldrich (St. Louis, MO, USA). ISRIB was purchased from Selleck Chemicals (Houston, TX, USA). Pan-Caspase inhibitor Q-VD-OPh was from Apexbio (Hsinchu, Taiwan). Antibodies against ATF4 (SC-200), GAPDH (SC-47724), α-tubulin (SC-23948), and MCL-1 (SC-819) were purchased from Santa Cruz Technology (Santa Cruz, CA, USA). Anti-TRAIL-R2/DR5 antibody (AF631) was obtained from R&D Systems (Minneapolis, USA). Anti-FLIP (7F10) (ALX-804–961) antibody was from Enzo Life Sciences (Farmingdale, NY, USA). Anti-caspase 8 antibody was generously provided by Dr. Gerald Cohen (Leicester University, UK). Antibodies against 4EBP1 (9452), p-4EBP1 (S65) (9451), AKT (9272), p-AKT (S473) (9271), ATF4 (D4B8) (11815), caspase-8 (1C12) (9746), CHOP (D46F1) (5554), eIF2α (D7D3) (5324), p-eIF2α (S51) (3597), and PERK (3192) were obtained from Cell Signaling Technology (CA, USA). Anti-Hsp70 (H5147) antibody was from Sigma-Aldrich. Horseradish peroxidase-conjugated secondary antibodies were from DAKO (P0447, P0448, P0449) (Cambridge, UK).

### Generation of stable cFLIP-overexpressing or cFLIP_L_-knockdown tumor cells by retro- or lentiviral infection

cFLIP_L_ and cFLIP_S_ (in pCR3.V64 vector, a kind donation of Dr. J. Tschopp, University of Lausanne) was cloned into *BamHI/SalI* and *HindIII/NotI* sites of retroviral vectors pBabe.puro and pLPCX, respectively. To obtain stably cFLIP_L_ knockdown cell lines, shRNA against cFLIP_L_ in a pSUPER vector (OligoEngine, Seattle, USA) was digested and cloned between *EcoR1* *and* *Cla1* into an H1 promoter-driven GFP-encoding pLVTHM lentiviral vector [44]. Retro- and lentiviruses were produced upon transfection of HEK293-T cells by the calcium phosphate method with the corresponding vectors. Retro- or lentiviruses-containing supernatants were collected 48 h after transfection and concentrated by ultracentrifugation at 22,000 rpm for 90 min at 4 °C. Tumor cells were plated at 6 × 10^5^ cells per 10-cm dish and infected with the viruses mentioned above 2 days later. Stable populations of tumor cells infected with retroviruses were obtained after selection in a culture medium containing puromycin (1.5 µg/ml) for 48 h. In order to obtain a population of stably cFLIP_L_ knockdown cells after lentiviral infection, infected cells were selected by GFP fluorescence using a BD FACSAria cell sorter (Becton-Dickinson, NJ, USA).

shRNAs sequences:

**Table:** 

cFLIP_L_	5′-GATCCCC**GAGCATACCTGAAGAGAGA**TTCAAGAGA**TCTCTCTTCAGGTATGCTC**TTTTTA-3′
Scrambled control	5′-GATCCCC**CTTTGGGTGATCTACGTTA**TTCAAGAGA**TAACGTAGATCACCCAAAG**TTTTTA-3′

### Spheroids

To generate spheroids, cells were seeded into Terasaki multiwell plates (Greiner Bio-One, Frickenhausen, Germany) (100 cells/well when HCT116 WT cells were employed and 400 cells/well in experiments performed with HCT116 *P53 KO* and HT-29 cells) and placed in humid chambers in the incubator. After 3 days of growth, spheres were transferred to 1.5% (w/v) agarose-coated F-bottom 96-well plates (Thermo Fisher Scientific, Roskilde, Denmark). The old medium was replaced with 50 µL of fresh medium every 2–3 days until spheroids reached a diameter of approximately 500 μm. Then, spheroids were treated as indicated in figure legends.

### Caspase-8 activity assay

To determine caspase-8 activity in 2D cell cultures, cells were seeded in 6-well plates as previously described. After treatment, cells were harvested, washed with phosphate-buffered saline (PBS), and resuspended in a temperate growth medium. To assess caspase-8 activity upon treatment in 3D cultures, spheroids were collected and dissociated using trypsin/EDTA. After dissociation, the trypsin reaction was stopped with growth media. Cells were washed once with PBS and resuspended in temperate growth media. Caspase-8 activity was determined by the Caspase-Glo^®^ 8 Assay in 2D and 3D cell extracts according to the manufacturer’s instructions (Promega, Madison, WI, USA). The luminescence intensity was analyzed in the Varioskan Flash microplate reader (Thermo Electron Corporation) after 90 min of incubation. Every condition was performed in duplicate.

### Analysis of hypodiploid apoptotic cells

Cells (1.5 × 10^5^/well) were cultured in 6-well plates and two days later treated as indicated in the figure legends. After treatment, hypodiploid apoptotic cells were detected by flow cytometry according to published procedures [45]. Briefly, cells were detached and dissociated with trypsin/EDTA and washed with cold PBS, fixed in 70% cold ethanol, and then stained with propidium iodide (40 μg/mL) while treating with RNAse (100 μg/mL). In order to analyze the subG1 population in 3D cultures, around 40 spheroids and their supernatants were collected and washed with PBS. After centrifugation, pelleted cells and spheroids were washed with temperate PBS and dissociated with trypsin/EDTA. Cells were fixed and stained as described before. Quantitative analysis of the cell cycle and subG1 cells was carried out in a FACSCalibur cytometer using the Cell Quest software (Becton Dickinson, Mountain View, CA, USA).

### Analysis of apoptosis by Annexin V-FITC/PI staining

Cells from 2D cultures or spheroids were washed with temperate PBS and stained with Annexin V-FITC (Immunostep, Salamanca, Spain) and propidium iodide (20 μg/mL, Sigma- Aldrich, MO, USA) in Annexin V binding buffer (10 mM HEPES/NaOH (pH 7.4), 140 mM NaCl, 2.5 mM CaCl_2_) for 15 min at room temperature in the dark, and then analyzed using a FACSCalibur cytometer (Becton Dickinson, Mountain View, CA, USA). Quantification of apoptotic cells was accomplished using Cell Quest software (Becton Dickinson, Mountain View, CA, USA).

### Protein synthesis

Global protein synthesis analysis was based on the SUnSET method [46] by puromycin immunodetection. Briefly, cells were stimulated as indicated and incubated in a culture medium containing puromycin at 1 μg/mL for 10 min. Then, cells were harvested for western blot analysis with an anti-puromycin antibody (clone 12d10) (Sigma-Aldrich, MO, USA).

### Immunoblot analysis of proteins

Cells were washed with PBS and lysed in TR3 lysis buffer (3% sodium dodecyl sulfate (SDS), 10% Glycerol, 10 mM Na_2_HPO_4_). Then, lysates were sonicated and protein content was measured with the Bradford reagent (Bio-Rad Laboratories, USA), before adding Laemmli sample buffer. Proteins were resolved on SDS–polyacrylamide minigels and detected as described previously [45]. GAPDH, α-tubulin and HSP70 were used as protein loading controls. Protein expression was analyzed using a ChemiDoc MP system and quantifications were performed with the Image Lab^TM^ 6.0 software (Bio-Rad Laboratories, Inc., CA, USA).

### RNA interference

siRNAs against cFLIP_L_, cFLIP_S_, cFLIP_L/S_, and non-targeting scrambled control oligonucleotides, were synthesized by Sigma (St. Louis, MO, USA). Cell suspensions of HCT116 cells were transfected with siRNAs using jetPRIME (Polyplus, Illkirch, France) as described by the manufacturer. After 24 h, the transfection medium was replaced with a regular medium, and cells were further incubated for 24 h before treatments.

siRNAs:

**Table:** 

cFLIP_L/S (DUAL)_;cFLIP_L_;cFLIP_S_	5′-GGGACCUUCUGGAUAUUUU[dt][dt]-3′; 5′-CCUAGGAAUCUGCCUGAUA[dt][dt]-3′; 5′-CACCCUAUGCCCAUUGUCC[dt][dt]-3′
Scrambled control	5′-CUUUGGGUGAUCUACGUUA[dt][dt]-3′

### Real time-qPCR

RNA was extracted using PRImeZOL (Canvax Biotech Córdoba, Spain) reagent, following the manufacturer’s instructions. mRNA expression was analyzed in triplicate by RT-qPCR on the ABI Prism7500 sequence detection system using predesigned assay-on-demand primers and probes (Applied Biosystems, Thermo Fisher Scientific, Roskilde, Denmark). Primers and probes used were: cFLIP_L_ (AIN1EV0), cFLIP_S_ (Ss03391532_m1) and Hypoxanthine-guanine phosphoribosyltransferase (HPRT1, Hs01003267_m1). HPRT was used as an internal control and mRNA expression levels were given as a fraction of mRNA levels in control cells.

### Statistical analysis

All data are presented as the mean ± standard deviation of at least three independent experiments. *P* < 0.05 was considered significant. Statistical analysis was performed using GraphPad Prism 8 (GraphPad Software, San Diego, CA, USA). The statistical test employed as indicated in figure legends.

## Supplementary information

Supplementary figure legends

Supplem. Figure S1

Supplem. Figure S2

Supplem. Figure S3

Supplem. Figure S4

Supplem. Figure S5

Supplem. Figure S6

Supplem. Figure S7

Supplem. Figure S8

Supplem. Figure S9

Supplem. Figure S10

Checklist

## Data Availability

All data generated or analyzed during this study are included in the main text and the supplementary information files.
