# Supplementary material for: cFLIP downregulation is an early event required for endoplasmic reticulum stress-induced apoptosis in tumor cells
Source: Cell Death Dis. 2022 Feb 3;13(2):111. doi: 10.1038/s41419-022-04574-6 (PMC8813907; doi:10.1038/s41419-022-04574-6)
Supplement: Supplementary file 1 — Supplementary figure legends [file 41419_2022_4574_MOESM1_ESM.docx]

**Supplementary figure legends**

**Figure S1. cFLIP down-regulation and PERK pathway activation upon treatment of HCT116 cells with either thapsigargin or tunicamycin.** HCT116 cells were treated with TG (100 nM) or TN (1 μg/mL) for the indicated times. eIF2α phosphorylation, CHOP, cFLIP and MCL-1 levels were determined in whole-cell extracts by western blotting. Levels of both FLIP isoforms, MCL-1 and CHOP werequantified using GAPDH as protein-loading control and referred to time 0h levels. eIF2α phosphorylation was quantified with respect to eIF2α total levels and referred to time 0h. Quantifications were performed with the Image Lab^TM^ 6.0 software.

**Figure S2. Apoptosis induction, cFLIP down-regulation, DR5 up-regulation and caspases activation in HCT116 *P53KO* cells upon ER stress. (A)** p53 depletion in HCT116 P53-null cells was confirmed by western blotting compared to WT cells. P53KO cells were treated or not with TG (100 nM) in the absence or presence of the pan-caspase inhibitor Q-VD-OPh (20 μM) for 48 h. Hypodiploid apoptotic cells were determined by flow cytometry. **(B and C)** HCT116 *P53KO* cells were treated with TG (100 nM) for the indicated times. cFLIP and CHOP levels **(B)** as well as TRAIL-R2/DR5 up-regulation, caspase-8 and caspase-3 processing **(C)** were determined in whole-cell extracts by western blotting. Levels of both cFLIP isoforms were quantified with Image Lab^TM^ 6.0 software using GAPDH as protein-loading control and graphed relative to time 0h levels. Blots are representative of three independent experiments.

**Figure S3. Apoptosis induction, cFLIP down-regulation and DR5 up-regulation in HT-29 cells upon ER stress. (A)** HT-29 cells were treated or not with TG (100 nM) in the absence or presence of the pan-caspase inhibitor Q-VD-OPh (20 μM) for 48 h. SubG1 cells were determined by flow cytometry. **(B and C)** HT-29 cells were treated with TG (100 nM) for the indicated times. cFLIP and CHOP levels **(B)** as well as TRAIL-R2/DR5 up-regulation **(C)** were determined in whole-cell extracts by western blotting. Relative levels of both cFLIP isoforms were quantified with Image Lab^TM^ 6.0 software using GAPDH as protein-loading control. Blots are representative of three independent experiments.

**Figure S4. ISRIB prevents cFLIP levels down-regulation.** In order to maintain protein synthesis upon ER stress, HCT116 cells were pre-treated with ISRIB (220 nM) for 1 h prior TG treatment for the indicated times. **(A)** To analyze protein synthesis, puromycin was added in the last 10 min of treatment and puromycin incorporation to the nascent protein chain was assessed by western blotting using an anti-puromycin antibody as described under Materials and Methods. **(B)** Levels of both cFLIP isoforms were determined in whole cell extracts by western blotting and quantified with Image Lab^TM^ 6.0 software using GAPDH as protein-loading control. Blots are representative of three independent experiments.

**Figure S5. Effect of ectopic expression of cFLIP on ER stress-induced activation of the PERK pathway (A)** HCT116 pBABE-ø, pBABE-cFLIP_L_, pLPCX-ø and pLPCX-cFLIP_S_ cells were treated with TRAIL (50 ng/mL) for 24 h and apoptosis was determined by subG1 analysis (**p ≤ 0.01; **** ≤ 0.0001; Two-way ANOVA. Tukey’s multiple comparisons test). **(B)** HCT116 WT, pBABE-ø and pBABE-cFLIP_L_ cells were treated or not with TG (100 nM) for the indicated times. cFLIP_L_, ATF4, CHOP and TRAIL-R2/DR5 protein levels were determined in whole-cell extracts by western blotting. GAPDH levels were used as protein-loading controls. Blots are representative of three independent experiments.

**Figure S6. Effect of cFLIP_L_ knockdown on ER stress-induced activation of the PERK pathway. (A)** HCT116 cells were either non-transfected (-) or transfected with a control oligonucleotide (Sc) or cFLIP_L_ siRNA (siFLIP_L_) for 48h prior to TG (100 nM) treatment for the indicated times. cFLIP, ATF4, CHOP and TRAIL-R2/DR5 protein levels were determined in whole-cell extracts by western blotting. GAPDH levels were used as protein-loading controls. Blots are representative of three independent experiments. **(B)** HCT116 cells grown as spheroids for 10 days were treated with or without TG (100 nM) in the presence or absence of pan-caspase inhibitor Q-VD-OPh (20 μM) for 4 days. Cell viability was analyzed by Annexin V-FITC and PI staining as described in the Material and Methods section. (**p ≤ 0.01; One-way ANOVA. Tukey’s multiple comparisons test).

**Figure S7**. **Activation of the PERK branch of the UPR, TRAIL-R2/DR5 up-regulation and cFLIP levels in HCT116 *P53KO* spheroids.** Cultures of HCT116 *P53KO* cells growing in 2D or as spheroids (3D) (8-days) were treated with TG (100 nM) for the indicated times. eIF2α phosphorylation, as well as ATF4, CHOP **(A)** and TRAIL-R2/DR5 **(B)** levels were assessed by western blotting. **(C)** cFLIP_L_ levels were also determined in whole-cell extracts by western blotting. Quantifications were performed using Image Lab^TM^ 6.0 software, by taking its respective untreated cultures as control at each time point. **(D)** 2D or 3D cultures of HCT116 *P53KO* cells were treated with or without TG (100 nM) for 24 h and caspase-8 activity measured as described in Material and Methods and graphed relative to 2D- or 3D-untreated conditions (*p ≤ 0.05. Multiple t-test. Holm-Sidak method).

**Figure S8**. **Activation of the PERK branch of the UPR, TRAIL-R2/DR5 up-regulation and cFLIP levels in HT-29 MCTSs.** Cultures of HT-29 cells growing in 2D or as spheroids (3D) (11-days) were treated with TG (100 nM) for the indicated times. PERK and eIF2α phosphorylation, as well as ATF4, CHOP **(A)** and TRAIL-R2/DR5 **(B)** levels were assessed by western blotting. **(C)** cFLIP_L_ levels were also determined in whole-cell extracts by western blotting. Quantifications were performed using Image Lab^TM^ 6.0 software.**(D)** 2D or 3D cultures of HT-29 cells were treated with or without TG (100 nM) for 48 h and caspase-8 activity measured by an enzymatic specific assay as described in Material and Methods and graphed relative to 2D- or 3D-untreated conditions (*p ≤ 0.05. Multiple t-test. Holm-Sidak method).

**Figure S9. Impact of mTORC1 inhibition on ER stress-induced changes in cFLIP levels and apoptosis in HCT116 cells. (A)** Cultures of HCT116 cells growing in 2D or as spheroids (3D) (10-days) were treated with TG (100 nM) for the indicated times. 4EBP1 phosphorylation was assessed by western blotting. **(B)** 2D cultures of HCT116 cells were pre-treated with 100 or 250 nM of Torin-1 (left panel) or 500 nM of Rapamycin (right panel) for 16 h, prior TG (100 nM) treatment for a further 2 h or 24 h. Torin-1 inhibitory action was assessed by western blot analysis of the phosphorylation of 4EBP1 and AKT, targets of mTORC1 and mTORC2, respectively. Likewise, rapamycin inhibitory effect on mTORC1 activity was determined by the loss of 4EBP1 phosphorylation. cFLIP levels and caspase-8 processing was also determined by western blotting. **(C)** 2D cultures of HCT116 cells were pre-treated with 100 or 250 nM of Torin-1 (left panel) or 500 nM of Rapamycin (right panel) for 16 h, prior TG (100 nM) treatment for 30 h and apoptosis was determined by subG1 analysis (****p ≤ 0.0001; Two-way ANOVA. Tukey’s multiple comparisons test).

**Figure S10**. **Effect of stable knockdown of cFLIP_L_ on ER stress-induced apoptosis in HCT116 cells. (A)** 2D cultures of shScr or shFLIP_L_ HCT116 cells were treated with TG (100 nM) or TRAIL (25 ng/mL) for 24 h and apoptosis was assessed by subG1 analysis (*** p ≤ 0.001; ****p ≤ 0.0001; Two-way ANOVA. Tukey’s multiple comparisons test). **(B)** cFLIP levels and caspase-8 processing were determined by western blotting in whole-cell extracts of shScr and shFLIP_L_ HCT116 cells treated with TG for 16 h. GAPDH was used as protein-loading control.
